# Sickness absence due to mental disorders among young adults: A register-based comparison of Finnish and Swedish speakers in Finland

**DOI:** 10.1177/14034948251360679

**Published:** 2025-08-07

**Authors:** Kaarina Reini, Kaija Appelqvist-Schmidlechner, Mika Gissler, Jan Saarela

**Affiliations:** 1 Demography Unit, Faculty of Education and Welfare Studies, Åbo Akademi University, Vaasa, Finland; 2 THL Finnish Institute for Health and Welfare, Department of Health Services, Prevention and Promotion, Tampere, Finland; 3 THL Finnish Institute for Health and Welfare, Department of Data and Analytics, Helsinki, Finland; 4 University of Turku, Research Centre for Child Psychiatry and Invest Research Flagship, Finland; 5 Region Stockholm, Academic Primary Health Care Centre, Stockholm, Sweden; 6 Karolinska Institutet, Department of Molecular Medicine and Surgery, Stockholm, Sweden

**Keywords:** Sickness allowance, mental health, young adults, ethnolinguistic groups, Finland

## Abstract

**Aims:**

Mental health problems of young adults are of increasing public health concern in the Nordic countries. The utilization of mental health services has been rising together with growing rates of sickness allowance due to mental health disorders. The aim of this study is to compare Finnish- and Swedish-speaking young adults in Finland and to examine their trends in sickness allowance receipt due to mental health problems in 2004–2018.

**Methods:**

We used register-based data and analysed people aged 20–34 years, distinguishing whether each person and the parents had Finnish or Swedish as their registered mother tongue. Cox regressions were used, in which the study outcome was first time receipt of sickness allowance due to mental disorders.

**Results:**

The hazard of sickness allowance receipt due to mental disorders increased for both Finnish and Swedish speakers during the study period. Swedish speakers started at a lower level, or about 0.8 that of Finnish speakers, but approached the level of Finnish speakers over the study period. Persons with bilingual background were largely found in between those with unilingual Finnish and unilingual Swedish background.

**Conclusions:**

**Before the coronavirus pandemic, the use of sickness allowance due to mental health problems increased and became more similar for Finnish- and Swedish-speaking young adults.**

## Background

**Figure 1.**
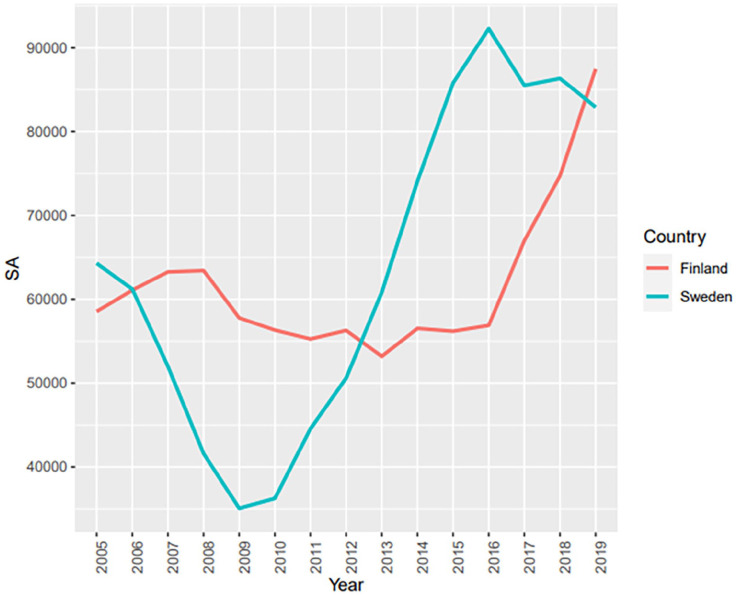
Prevalence of mental health-related sickness allowance receipt in Finland and Sweden. Numbers for Finland refer to started sickness allowance periods per year. Numbers for Sweden refer to sickness allowance (SA) periods that were valid at the end of the year. All age groups combined, for Finland persons aged 16–67 years and for Sweden persons 16 years and over. Data from Finland come from KELA [1]. Data from Sweden come from Försäkringskassan [2].

Mental health problems of young adults are of growing concern in the Nordic countries. The Finnish National Mental Health Strategy and Suicide Prevention Agenda 2020–2030 has declared the mental health situation in the daily lives of children and young people as one of its five priority areas. The increase of sickness allowance receipt due to mental disorders started in 2016 in Finland (Figure 1 [1,2]). This was several years later than in the neighbouring country Sweden, where the increase had begun already in the early 2010s, and tapered off around 2017. In both Finland and Sweden, the rise in mental health-related sickness absence was most prominent among young women aged 16–34 years, and the main causes were depression and anxiety [3,4]. Currently, mental health disorders constitute the leading cause for sickness allowance receipt among young people in Finland [5].

One mechanism behind the increase in sickness allowance receipt may be that changes in diagnostic practices have become more accurate in capturing mental health problems. Other, and related, explanations may be improved mental health literacy and diminished social stigma associated with the diagnoses [6]. Young people would then become more inclined to seek and receive treatment for mental problems, and regard them more as part of life and not as actual disorders [3]. A difference between personal expectations and a reality that does not measure up to the expectations might be another contributing factor [7]. Furthermore, social media encourages social comparisons and can be detrimental to self-esteem and mental health [8]. Substance use, social cohesion and mental health are also closely interrelated [9].

Variation in the prevalence of sickness allowance receipt across countries might consequently be due to various reasons, including differences in insurance schemes, legislation, macro-economic conditions, and cultural factors (Virtanen et al. [10] and references therein). In this study, we pay specific attention to the potential role of cultural factors. They might relate to how mental health is perceived, affect population subgroups at a different pace and result in different period effects. We focus on this notion by comparing Finnish and Swedish speakers in Finland.

Previous research has found that working-aged Swedish speakers have a lower prevalence of sickness allowance receipt than same-aged Finnish speakers, and that socioeconomic and demographic variables have modest influence on this difference [11]. Diagnosis-specific analysis has revealed that this language-group difference in sickness allowance receipt due to musculoskeletal and mental health has diminished over time and has suggested that more detailed analyses on young people, and particularly women, would be needed [12]. Cultural factors have been argued to underlie the overall better health position of Swedish speakers as compared with Finnish speakers in Finland [13,14].

Swedish speakers are generally also more proximate to Sweden and Swedish culture than Finnish speakers. Behind the linguistic facet that distinguishes Swedish and Finnish speakers in Finland, there are numerous identity markers related to group images, attitudes and social structure which separate the two groups and link Swedish speakers in Finland to Sweden [15]. It is reasonable to assume that influences from Sweden may affect the Swedish-speaking numerical minority in Finland. Examining a minority group with strong ties to another country may therefore shed light onto the issue of whether population health depends on cultural connections.

This study is the first to compare Finnish- and Swedish-speaking young adults in Finland with regard to sickness allowance receipt due to mental health disorders. In Finland, all non-retired persons aged 16–67 years are eligible for sickness allowance. Sickness allowance is paid after a waiting period of nine working days, and for a maximum period of approximately one calendar year. If the work incapacity continues thereafter, a person may apply for disability pension. Sickness allowance receipt can be considered an objective health measure, because it is conditional on a physician’s certificate that includes the main diagnosis for work incapacity according to the ICD classification (International Statistical Classification of Diseases and Related Health Problems) [16]. There is no national register on shorter sickness absence spells in Finland.

Our aim was to examine patterns of sickness allowance receipt due to mental disorders for Swedish and Finnish speakers over time in 2004–2018. We also extended our study to include the parents’ mother tongue. By doing so, we could evaluate whether persons with bilingual (Finnish and Swedish) background differ from those with unilingual Finnish and unilingual Swedish background, and whether own mother tongue matters within the group that has a bilingual background.

We expected to find a sharper increase in sickness allowance receipt due to mental health problems among Swedish speakers than among Finnish speakers during our study period, which would resemble the earlier increase in Sweden as compared with Finland. We also expected that own mother tongue is more important than parental mother tongue for sickness allowance receipt due to mental health problems, while persons with a bilingual background would lie in between those with unilingual Swedish and Finnish background (cf. Saarela and Kolk [13]).

## Methods

Our study design was nationwide longitudinal follow-up study of the years 2004–2018 based on register data. We used data from the Finnish population register [17] (FOLK data modules: Basic data, data on pairs of child–parents, Degree/Qualification, Migration), containing the entire resident population in Finland. We restricted our analyses to persons born in 1984–1996, meaning that they were aged 20–34 years during the study period. Each person was linked to data on sickness allowance receipt from the Social Insurance Institution of Finland (KELA) [18]. Data on SA receipt with information on diagnosis were unavailable before 2004.

All data access, data preparation and analyses were performed within Statistics Finland’s remote access system Fiona, with the contract number TK-52-694-18. The data were register-based and fully anonymised, and there was no need to seek specific separate ethical approval for the study from any other institutional review board than that at Statistics Finland. The requirement to obtain consent from the study persons was waived since no registered persons were contacted.

Each study person in the data could be linked to the mother and the father via an encrypted personal identification number. Data contain information about each person’s sex, year of birth, year of death, and the unique registered mother tongue (ethnolinguistic affiliation) as well as the mother tongue of each parent. We could therefore separate individuals according to their own and each parent’s mother tongue. Our analyses were restricted to Finnish- and Swedish-registered persons with Finnish- or Swedish-registered mothers and fathers. Foreign-born immigrants and people with a mother tongue other than Finnish or Swedish were beyond the scope of the paper. Figure S1 in the Supplementary material online describes how the study population was constructed.

### Statistical analysis

To compare Finnish and Swedish speakers with regard to sickness allowance receipt due to mental health problems, we applied Cox proportional hazards models. We ran models in which first-time sickness allowance receipt due to mental disorders constituted the time to event (response) variable. Mental disorders referred to all codes of the chapter F in the ICD-10 classification. The baseline hazard for sickness allowance receipt was time since age 20. Sickness allowance receipt before that age was rare. Right-censoring occurred at death, emigration, end of the observation period, or sickness allowance receipt due to a cause other than mental disorders. We ran separate models for men and women. For both sexes, the proportional hazards assumption was satisfactorily met.

In focus of the regressions was the interaction between (own/parental) mother tongue and observation year, and specifically how it related to sickness allowance receipt due to mental health disorders. Time-varying control variables measured at the beginning of each calendar year were educational level, family status and the share of Swedish speakers in the municipality of residence. Time-invariant control variables were mother’s and father’s educational level (the highest attained educational level), income quintile and labour market status, childhood family situation and whether the person lived in an owner-occupied dwelling. All time-invariant control variables except mother’s and father’s highest educational level were measured when the study person was aged 15 years. All variables were categorized (Table I, and Reini and Saarela [11]).

**Table I.** Characteristics of the study population.

Characteristics	Men	Women
**Number of persons**	408,365	390,830
**Number of person-years**	2,932,955	2,619,747
**Number of events** ^a^	22,949	37,506
	Person-years (%)	Person-years (%)
**Own mother tongue**		
Finnish	2,824,588 (94.5%)	2,554,992 (94.9%)
Swedish	165,138 (5.5%)	138,100 (5.1%)
**Own, mother’s, and father’s mother tongue** ^b^		
FFF	2,771,420 (92.7%)	2,508,880 (93.2%)
FSF/FFS	53,838 (1.8%)	47,103 (1.7%)
SSS	106,066 (3.5%)	86,636 (3.2%)
SSF/SFS	58,402 (2.0%)	50,473 (1.9%)
**Education**		
Primary	413,012 (13.8%)	267,648 (9.9%)
Secondary	2,158,325 (72.2%)	1,759,831 (65.3%)
Tertiary	418,389 (14.0%)	665,613 (24.7%)
**Family status**		
With partner and children	882,835 (29.5%)	667,438 (24.8%)
With partner, no children	740,702 (24.9%)	889,853 (33.0%)
Single	1,159,029 (38.8%)	936,391 (34.8%)
Other	207,160 (6.9%)	199,410 (7.4%)
**Childhood family situation**		
Both parents and sibling(s)	1,891,316 (63.3%)	1,709,677 (63.5%)
Both parents, no siblings	372,844 (12.5%)	324,308 (12.0%)
Mother and sibling(s)	321,165 (10.7%)	308,720 (11.5%)
Mother, no siblings	147,390 (4.9%)	147,564 (5.5%)
Father and sibling(s)	63,089 (2.1%)	46,807 (1.7%)
Father, no siblings	53,361 (1.8%)	29,398 (1.1%)
Other	140,561 (4.7%)	126,618 (4.7%)
**Mother’s labour market status**		
Employed	2,478,234 (82.9%)	2,240,759 (83.2%)
Unemployed	230,521 (7.7%)	202,072 (7.5%)
Outside labour market	252,634 (8.5%)	224,343 (8.3%)
Mother not present at age 15 years	28,337 (0.9%)	25,918 (1.0%)
**Father’s labour market status**		
Employed	2,472,803 (82.7%)	2,227,189 (82.7%)
Unemployed	207,605 (6.9%)	185,949 (6.9%)
Outside labour market	219,238 (7.3%)	197,560 (7.3%)
Father not present at age 15 years	90,080 (3.0%)	82,394 (3.1%)
**Mother’s income quintile**		
1st	563,970 (18.9%)	496,378 (18.4%)
2nd	626,314 (20.9%)	556,995 (20.7%)
3rd	635,293 (21.2%)	570,549 (21.2%)
4th	581,055 (19.4%)	535,016 (19.9%)
5th	554,757 (18.6%)	508,236 (18.9%)
Mother not present at age 15 years	28,337 (0.9%)	25,918 (1.0%)
**Father’s income quintile**		
1st	556,301 (18.6%)	497,221 (18.5%)
2nd	612,796 (20.5%)	544,004 (20.2%)
3rd	594,678 (19.9%)	534,340 (19.8%)
4th	580,237 (19.4%)	526,110 (19.5%)
5th	555,634 (18.6%)	509,023 (18.9%)
Father not present at age 15 years	90,080 (3.0%)	82,394 (3.1%)
**Mother’s highest education**		
Primary	319,185 (10.7%)	281,820 (10.5%)
Secondary	1,382,916 (46.3%)	1,235,364 (45.9%)
Tertiary	1,287,625 (43.1%)	1,175,908 (43.7%)
**Father’s highest education**		
Primary	557,771 (18.7%)	497,736 (18.5%)
Secondary	1,406,277 (47.0%)	1,260,106 (46.8%)
Tertiary	1,025,678 (34.3%)	935,250 (34.7%)
**Owner-occupied dwelling**		
Yes	2,381,705 (79.7%)	2,140,573 (79.5%)
No	608,021 (20.3%)	552,519 (20.5%)
**Year**		
2004	31,932 (1.1%)	30,704 (1.1%)
2005	61,646 (2.1%)	58,869 (2.2%)
2006	89,190 (3.0%)	84,637 (3.1%)
2007	114,891 (3.8%)	108,689 (4.0%)
2008	140,818 (4.7%)	132,786 (4.9%)
2009	165,455 (5.5%)	155,149 (5.8%)
2010	190,678 (6.4%)	177,334 (6.6%)
2011	214,443 (7.2%)	198,132 (7.4%)
2012	237,551 (7.9%)	217,302 (8.1%)
2013	258,482 (8.6%)	234,310 (8.7%)
2014	278,674 (9.3%)	250,123 (9.3%)
2015	297,222 (9.9%)	263,989 (9.8%)
2016	314,381 (10.5%)	276,071 (10.3%)
2017	302,780 (10.1%)	260,206 (9.7%)
2018	291,583 (9.8%)	244,791 (9.1%)
**Percentage Swedish speakers in municipality**		
<0.40%	1,246,076 (41.7%)	1,063,104 (39.5%)
0.40–1.49%	655,489 (21.9%)	569,094 (21.1%)
1.50–9.99%	871,977 (29.2%)	885,868 (32.9%)
10.00–29.99%	90,804 (3.0%)	75,737 (2.8%)
30.00–49.99%	45,462 (1.5%)	38,479 (1.4%)
50.00–74.99%	41,084 (1.4%)	32,292 (1.2%)
⩾75.00%	38,834 (1.3%)	28,518 (1.1%)

a Number of events is the number of first-time sickness allowance receipt due to mental disorders.

b First letter refers to own mother tongue (Finnish (F) or Swedish (S)), second letter to mother’s mother tongue (Finnish or Swedish), and third letter to father’s mother tongue (Finnish or Swedish).

Estimates are presented as hazard ratios with 95% confidence intervals. A hazard ratio of 1.20, for instance, means that the probability of an event, in this case sickness allowance receipt, at time *t* is 20% higher in one group as compared with that in another (the reference) group. R 3.2.2 was used in all analyses.

## Results

Table I gives variable distributions for the applied variable categorizations by sex. The study population consisted of 390,830 women and 408,365 men. The numbers of first-time sickness allowance recipients due to mental health disorders were 37,506 for women and 22,949 for men. Almost 95% of the study persons had Finnish as their mother tongue, and close to 93% were Finnish speakers with unilingual Finnish background.

**Figure 2.**
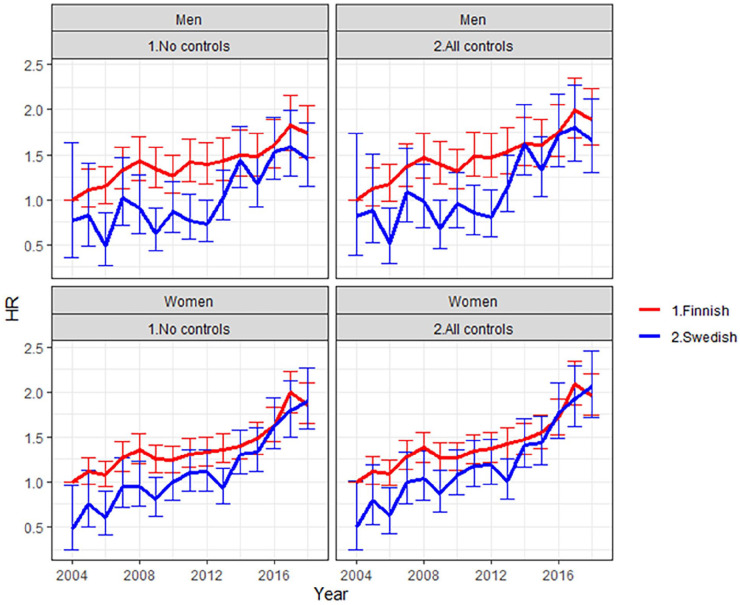
Hazard ratio of mental health-related sickness allowance receipt by mother tongue in the period 2004–2018. Finnish speakers in 2004 have been set to 1. Whiskers indicate 95% confidence intervals. ‘No controls’ refers to unadjusted regressions and ‘All controls’ to regressions adjusted for all control variables presented in Table I. The hazard ratios (HR) and confidence intervals in numeric format can be found in Table S1A and Table S1B in the Supplementary material online.

Figure 2 presents hazard ratios of sickness allowance receipt due to mental disorders, for men and women, respectively, based on regressions with a joint variable of own mother tongue and observation year. The upper panel is for men and the lower is for women. Results from the unadjusted model (Figure 2, left-hand side) and the adjusted model (right-hand side) are similar, so we will here focus on the latter. Figure 2 illustrates the trends over time in mental health-related sickness allowance receipt, where Finnish speakers in 2004 has been set to 1. Figure S2 in the Supplementary material gives the difference between Finnish and Swedish speakers in each calendar year, based on the same regressions, but where Finnish speakers have been set to 1 for each year.

Figure 2 shows that the hazard of sickness allowance receipt increased for Finnish-speaking men, from 1 to 1.90, and for Swedish-speaking, from 0.82 to 1.67 (see Supplementary Table S1 for details). The increase was consequently even larger for Swedish speakers over the study period, and they approached the level of Finnish speakers. The difference between Finnish- and Swedish-speaking men was statistically significant only in 2006, 2009, 2011 and 2012 (Supplementary Figure S2). At the end of the study period, there was no statistically significant difference in sickness allowance receipt between the two groups (Supplementary Figure S2).

The patterns for women are similar to those for men. However, the increase in the hazard of sickness allowance receipt over time was even larger among Swedish-speaking women, from 0.50 to 2.05 over the study period, as compared with an increase from 1 to 1.95 for Finnish-speaking women (see Supplementary Table S1 for details). Also for women, the overall trend is that Swedish-speaking women approached the level of Finnish-speaking women, although in most years the difference between the two groups was statistically not significant (Supplementary Figure S2).

**Figure 3.**
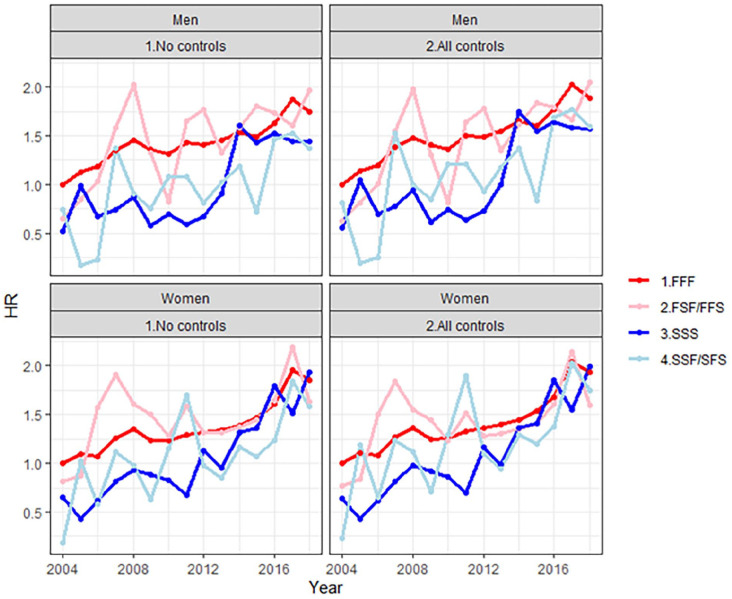
Hazard ratio of mental health-related sickness allowance receipt by own and parents’ mother tongue in the period 2004–2018. Finnish speakers with unilingual Finnish background have been set to 1. FFF, FSF, FFS, SSS, SSF, SFS: the first letter refers to own mother tongue (Finnish (F) or Swedish (S)), second letter to mother’s mother tongue (Finnish or Swedish) and third letter to father’s mother tongue (Finnish or Swedish). ‘No controls’ refers to unadjusted regressions and ‘All controls’ to regressions adjusted for all control variables presented in Table I. The hazard ratios (HR) and confidence intervals in numeric format can be found in Table S2A and Table S2B in the Supplementary material online.

Figure 3 presents results when also parental mother tongue was included, together with own mother tongue. Finnish speakers with unilingual Finnish background in 2004 was set to 1. Supplementary Figure S3 gives the difference as compared with Finnish speakers with unilingual Finnish background for each year, based on the same regressions.

Figure 3 shows that, for both sexes, the hazard of sickness allowance receipt increased in all groups. The increase for Finnish-speaking men with unilingual Finnish background was from 1 to 1.88, for Swedish-speaking men with unilingual Swedish background 0.56 to 1.57, for Finnish-speaking men with bilingual background 0.62 to 2.04, and for Swedish-speaking men with bilingual background 0.81 to 1.59 (see Supplementary Table S2 for details). Most of the differences as compared with Finnish-speaking men with unilingual Finnish background were statistically not significant (Supplementary Figure S3). For women in the corresponding groups, the increase was 1 to 1.93, 0.64 to 1.99, 0.77 to 1.60, and 0.23 to 1.75, respectively (Supplementary Table S2). Also for women, few of the differences as compared with Finnish speakers with unilingual Finnish background were statistically significant (Supplementary Figure S3).

For both sexes, the overall trend in sickness allowance receipt was nevertheless that the four groups approached each other over time, at least for Swedish- and Finnish-speaking women with unilingual background, while for men this pattern was not equally clear. The pattern for persons with bilingual background was rather erratic owing to the small number of observations but suggests that they are positioned in between those with unilingual Finnish and unilingual Swedish background. Finnish speakers with bilingual background were in general positioned somewhat closer to Finnish speakers with unilingual Finnish background, and Swedish speakers with bilingual background somewhat closer to Swedish speakers with unilingual Swedish background. However, there was considerable variation in the point estimates, and no firm conclusions could be made on this point.

## Discussion

We have studied trends in young adults’ mental health-related sickness allowance receipt by own and parental mother tongue in Finland in 2004–2018. At the beginning of the study period, the hazard was lower among people with Swedish mother tongue and unilingual Swedish background as compared with persons with Finnish mother tongue and unilingual Finnish background. In all groups and for both sexes, the hazard increased notably over time, but the differences between the language groups diminished.

Many factors may have contributed to the observed increase in sickness allowance receipt due to mental disorders among young adults in Finland. One is an intensified working life, making it harder to balance work, family and social commitments. It is known that sickness absence is associated with poor work–life balance and persons’ position in the labour market [19–21]. This may concern particularly women, as they more often face a combined work–family burden [22]. Another contributing factor may be the increased recognition of mental health problems and diminished stigma of mental disorders [23]. The level of stigma also seems to be lower among younger than older people [24] and personal stigma, rather than perceived public stigma, may be more decisive for seeking treatment [25].

Perhaps most noteworthy still is that we observed a steeper increase in the hazard of mental health-related sickness allowance receipt for Swedish speakers than for Finnish speakers during a period when there was a steep increase also in Sweden. The temporal pattern observed for Swedish speakers in Finland thus reminds of that observed in Sweden, where the increase started earlier than in Finland (Figure 1), and it might therefore be culturally related. If Swedish speakers in Finland are more inclined and disposed to follow the trends in Sweden – in terms of the perception of mental health, health literacy and lower social stigma associated with mental health problems – the increase in sickness allowance receipt due to mental disorders may also occur earlier in time than for Finnish speakers.

Our findings thus suggest that trends in a neighbouring country may influence a minority group with strong cultural and social ties to that country. So even though the study reports an evaluation from a single Nordic country with limited applicability to other contexts, the results may be useful for studying trends in sickness absence across national or regional population subgroups that are culturally similar.

Some further support for the cultural argument was also found by distinguishing own and parental mother tongue. Own mother tongue was more important than parental mother tongue for sickness allowance receipt due to mental health problems. Persons with bilingual background were positioned in between those with unilingual Swedish and Finnish background. However, we could not firmly conclude that persons with bilingual background were closer to persons with unilingual background on the basis of own mother tongue.

The strength of this study lies in the extensive register-based data used, which cover the total population of Finland. Sickness allowance receipt was based on physicians’ assessments, minimizing memory flaws and selective reporting. The results are thus generalizable to the population of young adults.

The limitations lie in the issue of internal validity, meaning that we cannot be fully confident that the relationships established are not caused by other factors than those observed from the data. Health behaviours and health literacy, for instance, are two confounding factors that are both relevant for sickness allowance receipt, but information about them is not available in register data. It has been suggested that people with high health literacy report psychological symptoms less than those with low health literacy [26]. It is also possible that our study groups differ in health behaviours and health literacy, because in working ages, a higher proportion of Finnish speakers than Swedish speakers appear to be smokers, obese and physically inactive [27].

The rise in stress-related exhaustion is a key factor behind increased sickness absence due to mental disorders in Sweden (2010–2016), especially among women [28]. Exhaustion disorder was introduced into the Swedish ICD-10 in 2005, while not included in ICD-10 or ICD-11 outside Sweden [29]. We could not therefore analyse whether exhaustion disorder was a latent reason behind the increase in sickness allowance due to mental disorders in Finland, or whether Finnish and Swedish speakers differed in this respect.

In the Nordic countries, there is a strong discourse that young people may become whatever they want. High levels of social mobility, both upward and downward, may lead to a discrepancy between expectations and real-life experiences, and result in distress and poor mental health [7]. In the era of social media, unfavourable social comparisons are hard to avoid [8]. Neither has the mental health of young adults been immune to various modern stressors like climate change, depletion of natural resources, wars and the COVID-19 pandemic [30]. Such developments might also affect psychological well-being and have a harmful effect on mental health, which future studies might observe. Whether Finnish and Swedish speakers differ in these respects, and whether such differences vary over time, were research questions beyond this study.

## Conclusions

Before the COVID-19 pandemic, sickness allowance receipt due to mental disorders among young adults increased considerably in Finland, and the difference between Finnish and Swedish speakers diminished. In 2004 and the first years thereafter, Swedish speakers and those with unilingual Swedish background were found at a lower level of sickness allowance receipt than Finnish speakers and those with unilingual Finnish background, but after 2012 the difference narrowed rapidly. At the end of our study period, differences by own and parental mother tongue were small, and particularly modest among women. Future studies could tentatively explore the development observed here with more detailed causes – such as depression and anxiety – and extend to pandemic and post-pandemic periods.

## Supplemental Material

sj-docx-1-sjp-10.1177_14034948251360679 – Supplemental material for Sickness absence due to mental disorders among young adults: A register-based comparison of Finnish and Swedish speakers in Finland Supplemental material, sj-docx-1-sjp-10.1177_14034948251360679 for Sickness absence due to mental disorders among young adults: A register-based comparison of Finnish and Swedish speakers in Finland by Kaarina Reini, Kaija Appelqvist-Schmidlechner, Mika Gissler and Jan Saarela in Scandinavian Journal of Public Health

## References

[1] KELA. Statistical Database Kelasto. Sickness allowance, new payments, https://raportit.kela.fi/ibi_apps/WFServlet?IBIF_ex=NIT122ALYKIELI=E (accessed 22 January 2024).

[2] Försäkringskassan. Statistik inom sjukområdet: Startade sjukfall efter diagnos. [Statistics in the field of illness: Initiated illness cases after diagnosis.] In Swedish, https://www.forsakringskassan.se/statistik-och-analys/statistikdatabas#!/sjuk/sjp-startade-detaljerad-diagnos (accessed 22 January 2024).

[3] Lundin A Forsell Y Dalman C. Mental health service use, depression, panic disorder and life events among Swedish young adults in 2000 and 2010: A repeated cross-sectional study in Stockholm County, Sweden. Epidemiol Psychiatr Sci 2018;27:510-8. 10.1017/S2045796017000099 PMC6999011 28367775

[4] Blomgren J Perhoniemi R. Increase in sickness absence due to mental disorders in Finland: Trends by gender, age and diagnostic group in 2005–2019. Scand J Public Health 2022;50:318-22. 10.1177/1403494821993705 PMC9096587 33615899

[5] KELA. Information package: Sickness absence, https://tietotarjotin.kela.fi/en/information-package/2699253/information-package-sickness-absence (accessed 16 June 2023).

[6] Wiens K Bhattarai A Pedram P , et al. A growing need for youth mental health services in Canada: Examining trends in youth mental health from 2011 to 2018. Epidemiol Psychiatr Sci 2020;29:e115:1-9. 10.1017/S2045796020000281 PMC7214527 32299531

[7] Jacobsen B Nørup I. (2020). Young people’s mental health: Exploring the gap between expectation and experience. Educ Res 2020;62:249-65.

[8] Braghieri L Levy R Makarin A. Social media and mental health. Am Econ Rev 2022;112:3660-93.

[9] Tsai DH Foster S Gmel G , et al. Social cohesion, depression, and substance use severity among young men: Cross-sectional and longitudinal analyses from a Swiss cohort. Addict Behav 2020;110:106510. 32623236 10.1016/j.addbeh.2020.106510

[10] Virtanen P Vahtera J Nygård CH. Locality differences of sickness absence in the context of health and social conditions of the inhabitants. Scand J Public Health 2010;38:309-16. 10.1177/1403494809364561 20435618

[11] Reini K Saarela J. Differences in sickness allowance receipt between Swedish Speakers and Finnish speakers in Finland: A register-based study. Yearb Popul Res Finl 2017;52:43-58.

[12] Reini K Saarela J. Suomen- ja ruotsinkieliset sairauspäivärahan saajat sairausryhmittäin – Kehityssuuntien tarkastelu 2005–2018. [Finnish and Swedish speaking sickness allowance receivers according to diagnostic group – trends in 2005–2018]. In Finnish. Lääkärilehti 2022;77:e323.

[13] Saarela J Kolk M. Alcohol-related mortality by ethnic origin of natives: A prospective cohort study based on multigenerational population register data from Finland and Sweden. BMJ Open 2020; 10:e042234. 10.1136/bmjopen-2020-042234 PMC7682461 33444215

[14] Paljärvi T Suominen S Koskenvuo M , et al. The differences in drinking patterns between Finnish-speaking majority and Swedish-speaking minority in Finland. Eur J Public Health 2009;19:278-84. 10.1093/eurpub/ckp007 19208699

[15] Saarela J Scott K. Naturalization in a context of free mobility: Evidence from cross-national data on Finnish immigrants in Sweden. Eur J Popul 2020;36:317-35. 10.1007/s10680-019-09530-3 PMC7113355 32256261

[16] World Health Organization. International Classification of Diseases (ICD), https://www.who.int/standards/classifications/classification-of-diseases (accessed 5 April 2024).

[17] Statistics Finland. Taika catalogue on research data modules, https://taika.stat.fi/en/ (accessed 4 April 2024).

[18] KELA. Description of data: Paid sickness allowance periods, https://aineistokatalogi.fi/catalog/studies/8000fcf8-a03b-49c4-97e2-98304f483fbd/datasets/ae8e0832-e8cb-48f9-9c72-46ddc779fe11 (in Finnish) (accessed 4 April 2024).

[19] Antai D Oke A Braithwaite P , et al. A ‘balanced’ life: Work–life balance and sickness absence in four Nordic countries. Int J Occup Environ Med 2015;6:205–22. 10.15171/ijoem.2015.667 PMC6977043 26498049

[bibr20-14034948251360679] Jansen NW Kant IJ van Amelsvoort LG , et al. Work–family conflict as a risk factor for sickness absence. Occup Environ Med 2006;63:488–94. 10.1136/oem.2005.024943 PMC2092520 16698806

[21] Pichler S. Sickness absence, moral hazard, and the business cycle. Health Econ 2015;24:692-710. 24737552 10.1002/hec.3054

[22] Nilsen W Skipstein A Østby KA , et al. Examination of the double burden hypothesis – a systematic review of work – family conflict and sickness absence. Eur J Public Health 2017;27:465–71. 10.1093/eurpub/ckx054 PMC5445721 28486653

[23] Filatova S Upadhyaya S Kronström K , et al. Time trends in the incidence of diagnosed depression among people aged 5–25 years living in Finland 1995–2012. Nord J Psychiatry 2019;73:475-81. 10.1080/08039488.2019.1652342 31443615

[24] Aromaa E Tolvanen A Tuulari J , et al. Predictors of stigmatizing attitudes towards people with mental disorders in a general population in Finland. Nord J Psychiatry 2011;65:125-32. 10.3109/08039488.2010.510206 20735187

[25] Golberstein E Eisenberg D Gollust SE. Perceived stigma and mental health care seeking. Psychiatr Serv 2008;59:392-9. 10.1176/ps.2008.59.4.392 18378838

[26] Paasio H Roos E Paakkari L , et al. Hälsolitteracitet och dess samband med motions- och idrottsvanor och subjektiv hälsa bland finlandssvenska studerande på andra stadiet. [Health literacy and its associations with physical activity, sports club participation, and subjective health among Swedish-speaking Finns in upper secondary schools and vocational schools.] In Swedish. Sos Laaketiet Aikak 2023;60:406-22.

[27] Andersén H Kankaanranta H Tuomisto LE , et al. Multimorbidity in Finnish and Swedish speaking Finns; association with daily habits and socioeconomic status – Nordic EpiLung cross-sectional study. Prev Med Rep 2021;22:101338. 33732608 10.1016/j.pmedr.2021.101338 PMC7937573

[28] Försäkringskassan. Psykisk ohälsa i dagens arbetsliv. [Mental illness in today’s working life.] In Swedish. Korta Analyser 2023:6.

[29] Lindsäter E Svärdman F Wallert J , et al. Exhaustion disorder: Scoping review of research on a recently introduced stress-related diagnosis. BJPsych Open 2022;8:e159. 10.1192/bjo.2022.559 PMC9438479 36458830

[30] Barchielli B Cricenti C Gallè F , et al. Climate changes, natural resources depletion, COVID-19 pandemic, and Russian–Ukrainian war: What is the impact on habits change and mental health? Int J Environ Res Public Health 2022;19:11929. 36231229 10.3390/ijerph191911929 PMC9565033

