# Supplementary material for: Sickness absence due to mental disorders among young adults: A register-based comparison of Finnish and Swedish speakers in Finland
Source: Scand J Public Health. 2025 Aug 7;54(6):615–23. doi: 10.1177/14034948251360679 (PMC13356260; doi:10.1177/14034948251360679)
Supplement: sj-docx-1-sjp-10.1177_14034948251360679 – Supplemental material for Sickness absence due to mental disorders among young adults: A register-based comparison of Finnish and Swedish speakers in Finland [file sj-docx-1-sjp-10.1177_14034948251360679.docx]

**Supplementary material**

**FIGURE S1:**

Cohorts born in 1984-1996,

living in Finland at age 20

N=850,740

(N(women)= 415,666; N(men)=435,074)

(FOLK Basic data)

Data on control variables and parents’ mother tongue

(FOLK Basic data, FOLK child-parents data, FOLK Degree/Qualification)

Data filtering: Own and parents’ mother tongue Finnish or Swedish, no disability pension before age 20

Sickness allowance data 2004-2018

(KELA SA data)

Data on disability pension receipt

(FOLK Basic data)

Data on emigrations 2004-2018 (FOLK Migration)

N= 850,740

N= 850,740

N= 850,740

N= 850,740

N= 799,195

N(women)=390,830

N(men)=408,365

TABLE S1A:

| Men |  | No controls | | All controls | |
| --- | --- | --- | --- | --- | --- |
| Mother tongue | Year | HR | 95% CI | HR | 95% CI |
| Finnish |  |  |  |  |  |
|  | 2004 | 1 |  | 1 |  |
|  | 2005 | 1.11 | 0.92, 1.34 | 1.12 | 0.93, 1.36 |
|  | 2006 | 1.15 | 0.96, 1.37 | 1.17 | 0.98, 1.40 |
|  | 2007 | 1.33 | 1.12, 1.58 | 1.36 | 1.15, 1.62 |
|  | 2008 | 1.43 | 1.21, 1.70 | 1.47 | 1.24, 1.74 |
|  | 2009 | 1.34 | 1.13, 1.59 | 1.39 | 1.18, 1.64 |
|  | 2010 | 1.27 | 1.07, 1.50 | 1.32 | 1.12, 1.56 |
|  | 2011 | 1.42 | 1.20, 1.67 | 1.49 | 1.26, 1.75 |
|  | 2012 | 1.39 | 1.18, 1.64 | 1.47 | 1.25, 1.74 |
|  | 2013 | 1.43 | 1.21, 1.68 | 1.53 | 1.30, 1.80 |
|  | 2014 | 1.50 | 1.27, 1.77 | 1.62 | 1.38, 1.92 |
|  | 2015 | 1.48 | 1.26, 1.74 | 1.60 | 1.36, 1.89 |
|  | 2016 | 1.61 | 1.36, 1.89 | 1.75 | 1.49, 2.06 |
|  | 2017 | 1.83 | 1.55, 2.16 | 1.99 | 1.69, 2.35 |
|  | 2018 | 1.74 | 1.47, 2.05 | 1.90 | 1.61, 2.24 |
| Swedish |  |  |  |  |  |
|  | 2004 | 0.76 | 0.36, 1.63 | 0.82 | 0.38, 1.74 |
|  | 2005 | 0.83 | 0.49, 1.40 | 0.88 | 0.52, 1.50 |
|  | 2006 | 0.48 | 0.27, 0.85 | 0.51 | 0.29, 0.91 |
|  | 2007 | 1.02 | 0.71, 1.47 | 1.09 | 0.75, 1.57 |
|  | 2008 | 0.90 | 0.63, 1.28 | 0.98 | 0.69, 1.39 |
|  | 2009 | 0.63 | 0.43, 0.91 | 0.68 | 0.46, 1.00 |
|  | 2010 | 0.87 | 0.64, 1.20 | 0.95 | 0.69, 1.31 |
|  | 2011 | 0.77 | 0.56, 1.06 | 0.85 | 0.62, 1.17 |
|  | 2012 | 0.73 | 0.53, 0.99 | 0.81 | 0.59, 1.11 |
|  | 2013 | 1.02 | 0.78, 1.33 | 1.14 | 0.87, 1.50 |
|  | 2014 | 1.44 | 1.14, 1.82 | 1.62 | 1.27, 2.06 |
|  | 2015 | 1.18 | 0.92, 1.50 | 1.32 | 1.03, 1.70 |
|  | 2016 | 1.53 | 1.22, 1.92 | 1.72 | 1.37, 2.17 |
|  | 2017 | 1.58 | 1.26, 1.99 | 1.80 | 1.43, 2.28 |
|  | 2018 | 1.46 | 1.15, 1.85 | 1.67 | 1.31, 2.13 |

TABLE S1B:

| Women | | | | No controls | | | | All controls | | | |
| --- | --- | --- | --- | --- | --- | --- | --- | --- | --- | --- | --- |
| Mother tongue | | Year | | HR | | 95% CI | | HR | | 95% CI | |
| Finnish | |  | |  | |  | |  | |  | |
|  | | 2004 | | 1 | |  | | 1 | |  | |
|  | | 2005 | | 1.11 | | 0.97, 1.27 | | 1.12 | | 0.98, 1.28 | |
|  | | 2006 | | 1.08 | | 0.95, 1.23 | | 1.09 | | 0.96, 1.24 | |
|  | | 2007 | | 1.27 | | 1.12, 1.44 | | 1.29 | | 1.13, 1.46 | |
|  | | 2008 | | 1.36 | | 1.20, 1.53 | | 1.38 | | 1.22, 1.55 | |
|  | | 2009 | | 1.25 | | 1.10, 1.41 | | 1.27 | | 1.13, 1.44 | |
|  | | 2010 | | 1.24 | | 1.10, 1.40 | | 1.27 | | 1.12, 1.43 | |
|  | | 2011 | | 1.31 | | 1.16, 1.48 | | 1.35 | | 1.20, 1.52 | |
|  | | 2012 | | 1.33 | | 1.18, 1.50 | | 1.37 | | 1.22, 1.54 | |
|  | | 2013 | | 1.36 | | 1.21, 1.53 | | 1.42 | | 1.26, 1.59 | |
|  | | 2014 | | 1.40 | | 1.25, 1.58 | | 1.47 | | 1.31, 1.66 | |
|  | | 2015 | | 1.48 | | 1.31, 1.66 | | 1.55 | | 1.38, 1.74 | |
|  | | 2016 | | 1.63 | | 1.45, 1.83 | | 1.71 | | 1.52, 1.92 | |
|  | | 2017 | | 1.99 | | 1.76, 2.23 | | 2.08 | | 1.85, 2.35 | |
|  | | 2018 | | 1.86 | | 1.65, 2.10 | | 1.95 | | 1.73, 2.20 | |
| Swedish | |  | |  | |  | |  | |  | |
|  | | 2004 | | 0.48 | | 0.24, 0.96 | | 0.50 | | 0.25, 1.02 | |
|  | | 2005 | | 0.75 | | 0.50, 1.13 | | 0.79 | | 0.52, 1.19 | |
|  | | 2006 | | 0.60 | | 0.41, 0.89 | | 0.63 | | 0.43, 0.93 | |
|  | | 2007 | | 0.95 | | 0.72, 1.27 | | 1.00 | | 0.75, 1.33 | |
|  | | 2008 | | 0.95 | | 0.73, 1.23 | | 1.03 | | 0.79, 1.34 | |
|  | | 2009 | | 0.81 | | 0.62, 1.05 | | 0.87 | | 0.67, 1.13 | |
|  | | 2010 | | 1.00 | | 0.80, 1.26 | | 1.08 | | 0.85, 1.36 | |
|  | | 2011 | | 1.10 | | 0.89, 1.36 | | 1.18 | | 0.95, 1.46 | |
|  | | 2012 | | 1.11 | | 0.90, 1.36 | | 1.19 | | 0.97, 1.47 | |
|  | | 2013 | | 0.93 | | 0.75, 1.15 | | 1.01 | | 0.81, 1.25 | |
|  | | 2014 | | 1.31 | | 1.09, 1.58 | | 1.41 | | 1.17, 1.71 | |
|  | | 2015 | | 1.33 | | 1.11, 1.60 | | 1.44 | | 1.19, 1.73 | |
|  | | 2016 | | 1.63 | | 1.37, 1.93 | | 1.76 | | 1.48, 2.10 | |
|  | | 2017 | | 1.79 | | 1.50, 2.12 | | 1.92 | | 1.61, 2.29 | |
|  | | 2018 | | 1.90 | | 1.59, 2.26 | | 2.05 | | 1.72, 2.46 | |


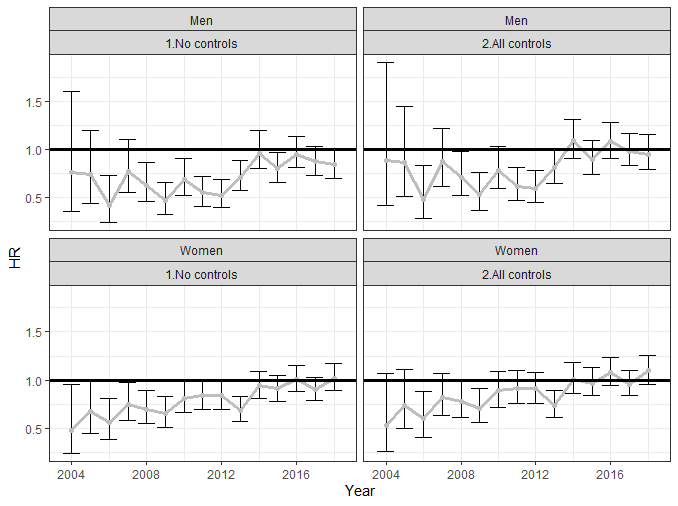
FIGURE S2:

TABLE S2A:

| Men |  | No controls | | All controls | |
| --- | --- | --- | --- | --- | --- |
| Own, mother's, father's  mother tongue | Year | HR | 95% CI | HR | 95% CI |
| **FFF** | 2004 | 1 |  | 1 |  |
|  | 2005 | 1.13 | 0.93, 1.37 | 1.14 | 0.94, 1.39 |
|  | 2006 | 1.18 | 0.98, 1.42 | 1.20 | 1.00, 1.44 |
|  | 2007 | 1.35 | 1.13, 1.62 | 1.38 | 1.16, 1.65 |
|  | 2008 | 1.45 | 1.22, 1.72 | 1.48 | 1.24, 1.76 |
|  | 2009 | 1.36 | 1.15, 1.62 | 1.41 | 1.18, 1.67 |
|  | 2010 | 1.31 | 1.10, 1.55 | 1.36 | 1.14, 1.61 |
|  | 2011 | 1.43 | 1.21, 1.70 | 1.50 | 1.26, 1.78 |
|  | 2012 | 1.41 | 1.19, 1.68 | 1.49 | 1.25, 1.77 |
|  | 2013 | 1.45 | 1.22, 1.72 | 1.54 | 1.30, 1.83 |
|  | 2014 | 1.53 | 1.29, 1.81 | 1.65 | 1.39, 1.95 |
|  | 2015 | 1.49 | 1.25, 1.76 | 1.60 | 1.35, 1.90 |
|  | 2016 | 1.63 | 1.37, 1.92 | 1.76 | 1.49, 2.09 |
|  | 2017 | 1.87 | 1.58, 2.21 | 2.02 | 1.71, 2.40 |
|  | 2018 | 1.74 | 1.46, 2.06 | 1.88 | 1.59, 2.24 |
| **FSF/FFS** |  |  |  |  |  |
|  | 2004 | 0.65 | 0.16, 2.64 | 0.62 | 0.15, 2.52 |
|  | 2005 | 0.85 | 0.35, 2.07 | 0.82 | 0.34, 1.99 |
|  | 2006 | 1.03 | 0.53, 2.02 | 1.01 | 0.52, 1.99 |
|  | 2007 | 1.58 | 0.97, 2.58 | 1.55 | 0.95, 2.53 |
|  | 2008 | 2.03 | 1.36, 3.05 | 1.98 | 1.32, 2.97 |
|  | 2009 | 1.32 | 0.83, 2.09 | 1.30 | 0.83, 2.06 |
|  | 2010 | 0.82 | 0.48, 1.40 | 0.81 | 0.48, 1.38 |
|  | 2011 | 1.65 | 1.14, 2.40 | 1.64 | 1.13, 2.39 |
|  | 2012 | 1.77 | 1.25, 2.51 | 1.78 | 1.25, 2.52 |
|  | 2013 | 1.32 | 0.90, 1.94 | 1.35 | 0.92, 1.98 |
|  | 2014 | 1.58 | 1.12, 2.24 | 1.62 | 1.14, 2.29 |
|  | 2015 | 1.80 | 1.30, 2.48 | 1.83 | 1.32, 2.54 |
|  | 2016 | 1.73 | 1.25, 2.39 | 1.79 | 1.29, 2.48 |
|  | 2017 | 1.60 | 1.14, 2.25 | 1.66 | 1.18, 2.33 |
|  | 2018 | 1.97 | 1.43, 2.72 | 2.04 | 1.48, 2.82 |
| **SSS** |  |  |  |  |  |
|  | 2004 | 0.52 | 0.17, 1.63 | 0.56 | 0.18, 1.76 |
|  | 2005 | 0.99 | 0.54, 1.83 | 1.05 | 0.57, 1.94 |
|  | 2006 | 0.67 | 0.36, 1.23 | 0.70 | 0.38, 1.30 |
|  | 2007 | 0.74 | 0.44, 1.24 | 0.78 | 0.46, 1.31 |
|  | 2008 | 0.87 | 0.56, 1.35 | 0.94 | 0.60, 1.46 |
|  | 2009 | 0.58 | 0.35, 0.94 | 0.62 | 0.38, 1.01 |
|  | 2010 | 0.69 | 0.45, 1.06 | 0.74 | 0.48, 1.14 |
|  | 2011 | 0.59 | 0.38, 0.91 | 0.64 | 0.41, 0.98 |
|  | 2012 | 0.67 | 0.45, 1.00 | 0.73 | 0.49, 1.09 |
|  | 2013 | 0.91 | 0.65, 1.28 | 0.99 | 0.70, 1.40 |
|  | 2014 | 1.60 | 1.22, 2.09 | 1.74 | 1.32, 2.30 |
|  | 2015 | 1.43 | 1.08, 1.88 | 1.55 | 1.16, 2.06 |
|  | 2016 | 1.52 | 1.16, 1.98 | 1.64 | 1.25, 2.16 |
|  | 2017 | 1.44 | 1.09, 1.91 | 1.58 | 1.19, 2.10 |
|  | 2018 | 1.44 | 1.08, 1.91 | 1.57 | 1.18, 2.11 |
| **SSF/SFS** |  |  |  |  |  |
|  | 2004 | 0.74 | 0.18, 2.97 | 0.81 | 0.20, 3.29 |
|  | 2005 | 0.17 | 0.02, 1.24 | 0.19 | 0.03, 1.37 |
|  | 2006 | 0.23 | 0.06, 0.92 | 0.26 | 0.06, 1.04 |
|  | 2007 | 1.37 | 0.82, 2.30 | 1.53 | 0.91, 2.56 |
|  | 2008 | 0.90 | 0.51, 1.58 | 0.99 | 0.56, 1.75 |
|  | 2009 | 0.75 | 0.43, 1.33 | 0.85 | 0.48, 1.50 |
|  | 2010 | 1.08 | 0.69, 1.70 | 1.21 | 0.77, 1.89 |
|  | 2011 | 1.08 | 0.71, 1.65 | 1.21 | 0.79, 1.86 |
|  | 2012 | 0.81 | 0.51, 1.28 | 0.92 | 0.58, 1.46 |
|  | 2013 | 1.02 | 0.68, 1.51 | 1.17 | 0.78, 1.74 |
|  | 2014 | 1.18 | 0.83, 1.70 | 1.37 | 0.95, 1.97 |
|  | 2015 | 0.72 | 0.47, 1.10 | 0.83 | 0.54, 1.28 |
|  | 2016 | 1.46 | 1.06, 2.00 | 1.69 | 1.23, 2.32 |
|  | 2017 | 1.52 | 1.11, 2.09 | 1.77 | 1.28, 2.43 |
|  | 2018 | 1.37 | 0.98, 1.91 | 1.59 | 1.14, 2.23 |

TABLE S2B:

| Women |  | No controls | | All controls | |
| --- | --- | --- | --- | --- | --- |
| Own, mother's, father's  mother tongue | Year | HR | 95% CI | HR | 95% CI |
| **FFF** | 2004 | 1 |  | 1 |  |
|  | 2005 | 1.10 | 0.96, 1.26 | 1.11 | 0.97, 1.27 |
|  | 2006 | 1.07 | 0.94, 1.22 | 1.08 | 0.94, 1.23 |
|  | 2007 | 1.26 | 1.11, 1.43 | 1.27 | 1.12, 1.44 |
|  | 2008 | 1.35 | 1.19, 1.53 | 1.36 | 1.20, 1.54 |
|  | 2009 | 1.23 | 1.09, 1.39 | 1.25 | 1.11, 1.42 |
|  | 2010 | 1.23 | 1.09, 1.39 | 1.26 | 1.11, 1.42 |
|  | 2011 | 1.29 | 1.14, 1.46 | 1.33 | 1.17, 1.50 |
|  | 2012 | 1.32 | 1.17, 1.49 | 1.36 | 1.20, 1.54 |
|  | 2013 | 1.34 | 1.19, 1.52 | 1.40 | 1.24, 1.58 |
|  | 2014 | 1.39 | 1.23, 1.57 | 1.45 | 1.28, 1.64 |
|  | 2015 | 1.47 | 1.30, 1.66 | 1.54 | 1.36, 1.74 |
|  | 2016 | 1.61 | 1.43, 1.81 | 1.68 | 1.49, 1.90 |
|  | 2017 | 1.96 | 1.73, 2.21 | 2.04 | 1.81, 2.31 |
|  | 2018 | 1.85 | 1.64, 2.09 | 1.93 | 1.71, 2.18 |
| **FSF/FFS** |  |  |  |  |  |
|  | 2004 | 0.82 | 0.34, 1.98 | 0.77 | 0.32, 1.86 |
|  | 2005 | 0.87 | 0.47, 1.64 | 0.84 | 0.45, 1.58 |
|  | 2006 | 1.57 | 1.04, 2.36 | 1.50 | 1.00, 2.26 |
|  | 2007 | 1.91 | 1.36, 2.68 | 1.84 | 1.31, 2.59 |
|  | 2008 | 1.61 | 1.15, 2.25 | 1.55 | 1.11, 2.16 |
|  | 2009 | 1.50 | 1.09, 2.08 | 1.44 | 1.04, 2.00 |
|  | 2010 | 1.28 | 0.92, 1.79 | 1.24 | 0.89, 1.72 |
|  | 2011 | 1.58 | 1.18, 2.11 | 1.52 | 1.14, 2.04 |
|  | 2012 | 1.32 | 0.97, 1.79 | 1.28 | 0.95, 1.74 |
|  | 2013 | 1.32 | 0.99, 1.77 | 1.30 | 0.97, 1.74 |
|  | 2014 | 1.37 | 1.04, 1.82 | 1.35 | 1.02, 1.79 |
|  | 2015 | 1.44 | 1.10, 1.89 | 1.41 | 1.07, 1.85 |
|  | 2016 | 1.64 | 1.27, 2.11 | 1.61 | 1.24, 2.08 |
|  | 2017 | 2.19 | 1.73, 2.77 | 2.14 | 1.69, 2.72 |
|  | 2018 | 1.63 | 1.24, 2.15 | 1.60 | 1.21, 2.11 |
| **SSS** |  |  |  |  |  |
|  | 2004 | 0.65 | 0.31, 1.38 | 0.64 | 0.3, 1.36 |
|  | 2005 | 0.43 | 0.22, 0.83 | 0.43 | 0.22, 0.83 |
|  | 2006 | 0.62 | 0.38, 0.99 | 0.62 | 0.38, 1.00 |
|  | 2007 | 0.81 | 0.55, 1.17 | 0.81 | 0.56, 1.19 |
|  | 2008 | 0.93 | 0.67, 1.28 | 0.98 | 0.71, 1.36 |
|  | 2009 | 0.89 | 0.66, 1.22 | 0.92 | 0.67, 1.26 |
|  | 2010 | 0.83 | 0.61, 1.12 | 0.86 | 0.63, 1.16 |
|  | 2011 | 0.68 | 0.49, 0.93 | 0.70 | 0.51, 0.97 |
|  | 2012 | 1.13 | 0.88, 1.45 | 1.17 | 0.91, 1.51 |
|  | 2013 | 0.95 | 0.73, 1.23 | 0.99 | 0.76, 1.29 |
|  | 2014 | 1.32 | 1.05, 1.65 | 1.36 | 1.08, 1.72 |
|  | 2015 | 1.36 | 1.09, 1.70 | 1.41 | 1.12, 1.76 |
|  | 2016 | 1.80 | 1.48, 2.19 | 1.85 | 1.51, 2.27 |
|  | 2017 | 1.52 | 1.22, 1.89 | 1.55 | 1.24, 1.95 |
|  | 2018 | 1.94 | 1.58, 2.39 | 1.99 | 1.61, 2.47 |
| **SSF/SFS** |  |  |  |  |  |
|  | 2004 | 0.19 | 0.03, 1.40 | 0.23 | 0.03, 1.65 |
|  | 2005 | 1.02 | 0.56, 1.90 | 1.19 | 0.65, 2.16 |
|  | 2006 | 0.58 | 0.30, 1.10 | 0.65 | 0.34, 1.27 |
|  | 2007 | 1.12 | 0.73, 1.70 | 1.24 | 0.81, 1.90 |
|  | 2008 | 0.98 | 0.65, 1.50 | 1.12 | 0.74, 1.68 |
|  | 2009 | 0.63 | 0.40, 1.00 | 0.71 | 0.45, 1.14 |
|  | 2010 | 1.15 | 0.83, 1.60 | 1.29 | 0.93, 1.80 |
|  | 2011 | 1.70 | 1.30, 2.20 | 1.90 | 1.45, 2.48 |
|  | 2012 | 0.98 | 0.71, 1.40 | 1.09 | 0.79, 1.51 |
|  | 2013 | 0.85 | 0.61, 1.20 | 0.94 | 0.68, 1.31 |
|  | 2014 | 1.17 | 0.88, 1.50 | 1.29 | 0.98, 1.71 |
|  | 2015 | 1.07 | 0.81, 1.40 | 1.20 | 0.90, 1.58 |
|  | 2016 | 1.24 | 0.96, 1.60 | 1.38 | 1.07, 1.79 |
|  | 2017 | 1.84 | 1.47, 2.30 | 2.03 | 1.61, 2.54 |
|  | 2018 | 1.58 | 1.23, 2.00 | 1.75 | 1.37, 2.24 |


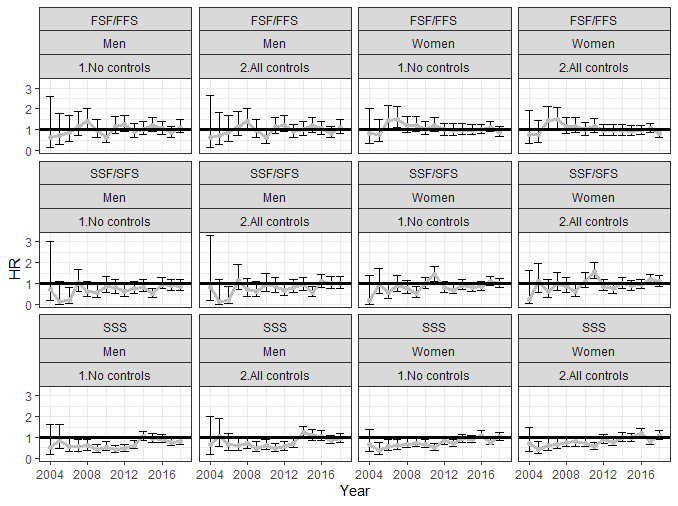
FIGURE S3:
